# Use of flash glucose-sensing technology in patients with type 2 diabetes treated with liraglutide combined with CSII: a pilot study

**DOI:** 10.1590/1414-431X20198652

**Published:** 2019-12-20

**Authors:** Ming-yan Yao, Li-qin Li, Jian-xia Ma, Peng Xue, Yu-kun Li

**Affiliations:** 1 Department of Endocrinology, The Third Hospital of Hebei Medical University, Shijiazhuang, China; 2 Department of Endocrinology, Baoding NO.1 Central Hospital, Baoding, Hebei, China

**Keywords:** Flash glucose monitoring, Glucagon-like peptide-1 analog, Continuous subcutaneous insulin infusion, Glucose fluctuation, Cardiometabolic risk markers, Type 2 diabetes

## Abstract

Glycemic variability (GV) may be linked to the development of diabetic complications by inducing inflammation, oxidative stress, and endothelial dysfunction. Flash glucose monitoring (FGM) provides a novel method of continuously monitoring interstitial glucose levels for up to 14 days. This study randomly assigned poorly controlled type 2 diabetes mellitus patients treated with metformin and multiple daily injections of insulin (n=60) to either continuous subcutaneous insulin infusion (CSII) treatment or CSII in combination with liraglutide (CSII+Lira) treatment for 14 days during hospitalization. GV was assessed using a FGM system; weight and cardiometabolic biomarkers were also evaluated. The coefficient of variation was significantly reduced in the CSII+Lira group (P<0.001), while no significant change was observed in the CSII group. The changes differed significantly between the two groups in mean amplitude of glycemic excursions (P=0.004), standard deviation (P=0.006), and the percentage of time in the target range (4–10 mmol/L, P=0.005 and >10 mmol/L, P=0.028). The changes in mean of daily differences, interquartile range, and percentage of time in hypoglycemia (<3.3 mmol/L) and hyperglycemia (>13.9 mmol/L) identified by FGM showed no difference. Treatment with liraglutide increased serum adiponectin [33.5 (3.5, 47.7) pg/mL, P=0.003] and heme oxygenase-1 levels [0.4 (–0.0, 1.8) ng/mL, P=0.001] and reduced serum leptin levels [–2.8 (3.9) pg/mL, P<0.001]. Adding the glucagon-like peptide-1 analog liraglutide improved GV, weight, and some cardiometabolic risk markers. The FGM system is, therefore, shown to be a novel and useful method for glucose monitoring.

## Introduction

Recent evidence has demonstrated that insulin has an anti-oxidative and anti-inflammatory action in humans (1). Intensive insulin therapy has been shown to reduce the risk of microvascular complications in the short-term and macrovascular complications after 10 years of post-trial follow-up in newly diagnosed patients with type 2 diabetes (2–4). Short-term intensive insulin treatment could effectively optimize the sugar control profile and improve β-cell function (5). Although an effective treatment for hyperglycemia, insulin may be associated with an increased risk of weight gain and hypoglycemia. Blood glucose monitoring is critical for effective diabetes management (6). Self-monitoring of blood glucose (SMBG), continuous glucose monitoring systems (CGMSs), and glycated hemoglobin (HbA1c) are the most commonly used methods to assess short-term and long-term blood glucose control. Although SMBG has been shown to improve glycemic control when used within a structured testing regimen, it cannot predict impending hypoglycemia or alert the patient to a current hypoglycemic state. CGMSs can record real-time glycemic values and trends by providing a large number of blood glucose recordings (7). However, they have not been widely used because of device limitations such as short sensor lifetimes and the need for SMBG for device calibration. HbA1c could assess glycemic control in 8–12 weeks. However, some studies have suggested that targeting HbA1c may not always result in cardiovascular benefit (8), which emerged in only one study during passive follow-up (9). One underlying reason for this discrepancy is that HbA1c cannot reflect glycemic variability (GV), which is mainly due to progressive insulin deficiency, which increases with duration of type 2 diabetes.

GV can be simply defined as the degree to which fluctuations of glucose between high (peaks) and low (nadir) levels exist. Wide fluctuations in blood glucose levels can induce oxidative stress, inflammation, and endothelial dysfunction, which can be linked to the development of cardiovascular complications (10). Furthermore, GV is associated with both microvascular and macrovascular endpoints in type 2 diabetes (11). Flash glucose monitoring (FGM) system by FreeStyle Libre (Abbott Diabetes Care Inc., USA) has provided a new method of quantifying glycemia and GV. The sensor is applied to the skin with a handheld applicator and lasts for 14 days. In addition to updating current glucose levels in real time, FreeStyle Libre system also uses trend arrows to provide information on the direction and rate of change in glucose levels. It is worth noting that SMBG calibration is not required during the 14-day wearing period.

Some studies have shown the accuracy and safety of FGM systems (12). However, using a FGM system to investigate glycemic control and GV in poorly controlled type 2 diabetes mellitus (T2DM) treated with glucagon-like peptide-1 (GLP-1) analog in combination with continuous subcutaneous insulin infusion (CSII) therapy is rare. It has been demonstrated that CSII is associated with lower glucose variability and overall hypoglycemic events than multiple daily injections of insulin (MDI) in young adults with type 1 diabetes (13). Furthermore, GLP-1 could improve endothelial function and influence various cellular pathways including anti-inflammatory and anti-oxidative stress pathways (14,15). Based on this evidence, the therapeutic option of combining CSII and GLP-1 may have clinical benefit. In this study, we used FreeStyle Libre Pro (FSL-pro) for FGM to obtain blinded ambulatory glucose profile (AGP) data on blood glucose over a 14-day period at multiple time-points, and measured the levels of serum cardiometabolic risk markers at baseline and endpoint.

## Material and Methods

### Design and study population

A run-in procedure was used to establish a stable baseline, familiarize candidates with procedures, and identify those unable to adhere to study requirements. The candidates received metformin and MDI (basal-bolus insulin+rapid-acting insulin analogs or premixed insulins), without any other glucose-lowering medication. Use of metformin was required with the dosage increasing from 500 mg daily to 2,000 mg daily, as tolerated, and the dosage had to have been adjusted to 2,000 mg daily prior to hospital admission. Patients who could not tolerate 2000 mg metformin daily were excluded. During this period, candidates performed SMBG provided by our study. The baseline characteristics of the 60 recruited individuals with T2DM are shown in Table 1. The other inclusion criteria were: a) age >20 years; b) a diagnosis of T2DM for ≥1 year; c) received metformin and MDI for at least 2 months, without any other glucose-lowering medication; d) HbA1c between 8 and 14%; e) fasting C-peptide ≥0.5 mg/mL (0.17 nmol/L); and f) body mass index (BMI) not higher than 45 kg/m^2^.

**Table 1:** Baseline characteristics of diabetes mellitus patients under continuous subcutaneous insulin infusion (CSII) and CSII+liraglutide (Lira) treatments.

Characteristic	CSII+Lira	CSII	P value
Number	30	30	
Gender (female/male)	3/27	7/23	0.166
Age	50.0 (42.5, 63.5)	49.0 (42.5, 61.0)	0.773
Diabetes mellitus duration (years)	9.0 (2.0, 17.5)	6.0 (3.0, 13.0)	0.452
Blood pressure (mmHg)			
SBP	140.0 (18.1)	135.3 (17.4)	0.313
DBP	94.3 (12.0)	94.1 (13.5)	0.952
Weight (kg)	85.5 (9.8)	82.4 (11.8)	0.283
BMI (kg/m2)	29.4 (3.4)	29.0 (4.2)	0.745
FPG (mmol/L)	9.9 (1.0)	9.5 (0.9)	0.088
HbAIC (%)	9.3 (8.5,13.0)	10.0 (8.8,12.0)	0.635
Family history (yes/no)	15/15	14/16	0.796
Smoking habit (yes/no)	11/19	8/22	0.405
Hypertension (yes/no)	19/11	18/12	0.371
Dyslipidemia (yes/no)	20/10	17/13	0.426
TG (mmol/L)	1.6 (1.1, 4.2)	2.0 (1.4, 4.7)	0.530
TC (mmol/L)	5.0 (1.7)	5.0 (1.7)	0.892
LDL-c (mmol/L)	2.9 (1.1)	2.9 (1.2)	0.862
HDL-c (mmol/L)	1.2 (1.1, 1.4)	1.2 (1.1, 1.4)	0.784
FC-P (ng/mL)	2.1 (1.4)	2.0 (1.3)	0.879
TSH (uIU/mL)	1.4 (1.1, 2.9)	1.6 (1.1, 2.9)	0.574
FT3 (nmol/L)	5.1 (4.9, 5.4)	5.1 (4.8, 5.4)	0.529
FT4 (nmol/L)	13.9 (12.0, 15.4)	13.5 (10.0, 15.3)	0.558
hsCRP (mg/L)	1.9 (1.1, 4.7)	1.9 (1.0, 8.4)	0.287
Microalbuminuria (yes/no)	8/22	12/18	0.273
Cardiovascular disease (yes/no)	9/21	6/24	0.371
Anti-hypertensive therapies (yes/no)	17/13	18/12	0.793
Lipid-lowering therapies (yes/no)	20/10	19/11	0.787
Aspirin therapy (yes/no)	12/18	10/20	0.592

Data are reported as median (IQR), mean (SD), or number. SBP: systolic blood pressure; DBP: diastolic blood pressure; BMI: body-mass index; FPG: fasting plasma glucose; HbAIC: hemoglobin A1c; TG: triglycerides; TC: total cholesterol; LDL-c: low-density lipoprotein cholesterol; HDL-c, high-density lipoprotein cholesterol; FC-P: fasting C-peptide; TSH: thyroid-stimulating hormone; FT3: free thyroxine (T3); FT4: free thyroxine (T4); hsCRP: hypersensitive C-reactive protein.

The exclusion criteria were: a) inability or unwillingness to perform FGM; b) type 1 diabetes or gestational diabetes mellitus; c) severe acute or severe chronic diabetic complications; infection, recent trauma or surgery; d) renal dysfunction, defined as serum creatinine level ≥1.5 mg/dL for males or ≥1.4 mg/dL for females; e) liver dysfunction, defined as alanine aminotransferase or aspartate aminotransferase levels three or more times the upper limit of normal; f) serious cardiovascular disorders and current symptomatic heart failure; and g) pregnant or lactating women, or a history of pancreatitis.

This randomized, open-labeled and parallel-design study consisted of 14-day treatment periods. Subjects who were inadequately controlled with metformin and MDI were randomly assigned (through a computer-generated, random order) 1:1 to one of two treatment groups: control group (CSII group) or CSII in combination with liraglutide (CSII+Lira group). All participants received metformin 2,000 mg daily and stopped receiving MDI treatment within this period. During hospitalization, patients in the CSII+Lira group received 0.6 mg liraglutide (Victoza, Denmark) subcutaneously daily to begin with, which was then increased to either 1.2 or 1.8 mg daily in combination with CSII. The detailed procedure is as follows: liraglutide was injected starting at 0.6 mg/day; if there was no intolerance such as vomiting or nausea, the dose was increased every 3 days to 1.2 mg/day and then to 1.8 mg/day. Five patients had a transient loss of appetite at 0.6 mg/day. At 1.2 mg/day, 9 patients developed nausea and did not continue to increase the dose. At 1.8 mg/day, 1 patient developed vomiting and 1 patient developed diarrhea, however, the symptoms improved after the dosage was returned to 1.2 mg/day. In summary, 11 subjects (37%) were on 1.2 mg liraglutide per day, and 19 (63%) were on 1.8 mg/day. The average dose of liraglutide used in the CSII+Lira group was 1.58 mg/day. The period of time in the study considered as the patient being on a stable dose of liraglutide was 7–11 days. All patients in the CSII+Lira group used FSL-pro for 14 days from the start of hospitalization. The CSII group received insulin aspart only (Novo Nordisk, Denmark) with an insulin pump (MMT-712EWS; Medtronic, USA). The initial insulin dose was 0.4–0.7 IU · kg^-1^ · day^-1^, with the total daily dose delivered as a 50/50 basal/bolus injections. All of the participants received a 14-day course of FGM during hospitalization. The final dose of insulin used in the 2 groups at the end of the 14 days was as follows: CSII group: 0.54 (0.5, 0.6) IU · kg^-1^ · day^-1^, and CSII+Lira group: 0.33 (0.30, 0.39) IU · kg^-1^ · day^-1^. The detailed dosages of insulin and liraglutide are shown in Table 2.

**Table 2:** Medication dosing of diabetes mellitus patients under continuous subcutaneous insulin infusion (CSII) and CSII+liraglutide (Lira) treatments.

Variable	CSII+Lira	CSII	P value
Initial dose of insulin (IU · kg^-1^ · day^-1^)	0.59 (0.55, 0.64)	0.62 (0.57, 0.65)	0.236
Final dose of insulin (IU · kg^-1^ · day^-1^)	0.33 (0.30, 0.39)	0.54 (0.50, 0.60)	0.000
Change in dose of insulin (IU · kg^-1^ · day^-1^)	0.25 (0.23, 0.28)	0.06 (0.04, 0.09)	0.000
1.2/1.8 mg liraglutide	11/19	−	
Average dose of liraglutide (mg/day)	1.58		

Data are reported as median (IQR).

This study was conducted at the Third Hospital of Hebei Medical University and was performed in accordance with the guidelines of the Declaration of Helsinki. All procedures were approved by the Ethical Committee of the Third Hospital of Hebei Medical University (CRT2018-015-1), and after being informed of the study procedures, all patients provided written informed consent before enrolling in the study. During hospital admission, the subjects received 1400–1800 kcal meals depending on their standard body weight.

### Endpoint

The primary endpoint of this study was the change in glycemic parameters during a 14-day period of hospitalization shown by FSL-pro. Secondary endpoints included weight and cardiometabolic biomarkers, which were measured under fasting conditions at baseline and 14 days after treatment.

### Flash glucose monitoring

The glycemic parameters of the subjects were provided by the FGM system, including mean blood glucose (MBG) levels, standard deviation (SD), mean amplitude of glycemic excursions (MAGE), mean of daily differences (MODD), the change from baseline to 14 days in the coefficient of variation (CV) of glucose values, the interquartile range (IQR), percentage of time in hypoglycemia (<3.3 mmol/L), time in target range (4–10 mmol/L), and in hyperglycemia (>13.9 mmol/L), as described previously (16,17). MAGE, MODD, and CV were used to assess blood glucose stability.

### Clinical and laboratory measurements

Gender, age, duration of diabetes, family history, smoking history, diabetic complications, hypertension history, and medication history were evaluated. Anthropometric measurements (height and weight) were obtained. Blood pressure was measured twice with the subjects in a seated position after 10 min of rest. BMI was calculated as weight (kg) divided by the square of height in meters (m^2^). Overweight and obesity were defined as 24≤ BMI <28 and BMI ≥28 kg/m^2^, respectively, using the Working Group on Obesity in China criteria (18).

Blood samples were obtained after a 12-h fast at baseline and after 14 days of liraglutide and CSII therapy. Serum and plasma were centrifuged (1000 *g* for 15 min at 4°C) within 30 min of collection and stored at -80°C. Measurements for biochemical markers, such as fasting plasma glucose (FPG), HbA1c, C-peptide, total cholesterol, low-density lipoprotein cholesterol, high-density lipoprotein cholesterol, triglycerides, hypersensitive C-reactive protein, and thyroid function were measured using an enzymatic method (Roche Diagnostics GmbH, Germany) in the laboratory of the Third Hospital of Hebei Medical University. We measured serum cardiometabolic biomarkers [8-iso prostaglandin F2α (8-iso-PGF2α), leptin, adiponectin, heme oxygenase-1 (HO-1), and interleukin (IL)-6] levels with an enzyme-linked immunosorbent assay (ELISA) kit.

### Statistical analysis

Data were analyzed with SPSS software for Windows version 22.0 (IBM, USA). The Kolmogorov-Smirnov normality test was used to analyze the distribution of variables. Normally distributed and continuous variables are reported as means±SD, and non-normally distributed variables are reported as median (interquartile range). Categorical variables are reported as proportions. The differences of the variables with a normal distribution between two groups were compared by independent *t*-tests, while the comparisons of non-normally distributed variables were tested using Mann-Whitney U-tests. The paired *t*-test or Wilcoxon rank sum test were performed to compare the changes before and after intervention in the same group. The chi-squared test or Fisher's exact test were applied to analyze the differences of proportions. A P-value of less than 0.05 was considered statistically significant (two-tailed).

## Results

### Participant flow and baseline characteristics

**Figure 1:**
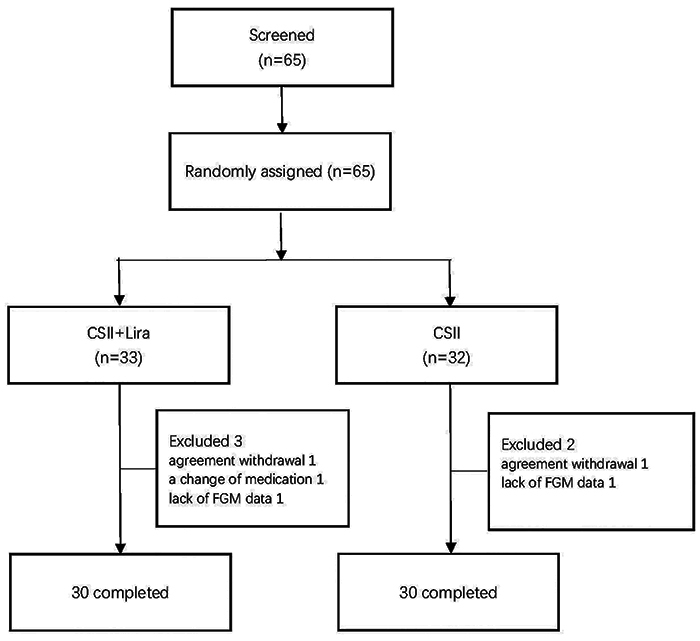
Flow chart of study participants. CSII: continuous subcutaneous insulin infusion; Lira: liraglutide; FGM: flash glucose monitoring.

The study design is illustrated in Figure 1. Sixty-five patients were randomized, and 60 patients completed the study and were included in this analysis. The baseline characteristics were balanced between the randomized groups (Table 1). Overall, median age was 49.5 (42.3, 61.0) years, median duration of diabetes was 7.0 (3.0, 14.0) years, and 25% had a prior cardiovascular event, which was either myocardial ischemia or coronary heart disease. At baseline, mean BMI was 29.2±3.7 kg/m^2^, mean FPG was 9.7±1.0 mmol/L, and median HbA1c level was 9.3% (8.6,12.0%). Microalbuminuria was present in 33% of participants. All 60 participants had a complete set of FGM data before and after 14 days of randomized treatment, allowing analysis for the primary endpoint.

### Changes in body weight

**Figure 2:**
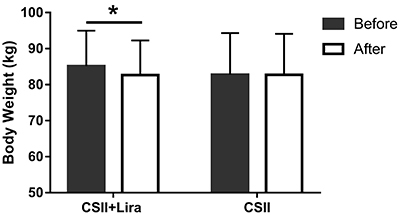
Change in weight (kg) at 14 days (After) compared with baseline (Before) in each treatment group. CSII: continuous subcutaneous insulin infusion; Lira: liraglutide; Before: before therapy (baseline); After: after 14 days of therapy. Data are reported as means±SD *P<0.05, after therapy compared to baseline (paired *t*-test).

Average weight at randomization was slightly higher in the CSII+Lira group than in the CSII group, 85.5±9.8 *vs* 82.4±11.8 kg, but without a statistical difference. Body weight decreased steadily in the CSII+Lira group with an average reduction of 2.5±0.5 kg (2.93% decrease compared to baseline, P<0.001), but slightly increased by 0.2±1.1 kg (0.24% increase compared to baseline, P=0.854) in the CSII group. The difference between the two groups was 2.7±0.2 kg (3.17%, P<0.001) after 14 days of treatment, (Figure 2).

### Changes in glucose profiles measured by FSL-pro

**Figure 3:**
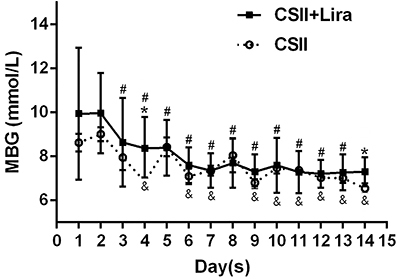
Mean blood glucose (MBG) values during a 14-day hospitalization period of the two treatment groups. CSII, continuous subcutaneous insulin infusion; Lira, liraglutide. Data are reported as median (IQR). *P<0.05, between the CSII and CSII+Lira groups; ^#^P<0.05, MBG from different time points compared with day 1 baseline data in the CSII+Lira group and ^&^P<0.05, in the CSII group (Student's *t*-test).

The glycemic parameters of the subjects were measured using the FGM system FSL-pro. Overall, whether from MBG or some indicators of glycemic control, GV significantly improved after the liraglutide and CSII treatments (Figure 3 and Supplementary Table S1). After the 14-day hospitalization period, MBG, MAGE, MODD, SD, and IQR significantly improved in both treatment groups. Smaller decreases were found in MODD [–1.0 (–1.4, –0.6) *vs* –0.7 (–0.9, –0.5) mmol/L, P=0.129] and IQR [–1.6 (–2.8, –0.8) *vs* –0.9 (–1.6, –0.7) mmol/L, P=0.130] in the CSII+Lira group compared with the CSII group, although there were no statistically significant differences. CV, reported as the within-day standard deviation of the mean daily glucose, was significantly reduced by 4.5% in the CSII+Lira group (P<0.001). In contrast, there was no significant change in the CSII group (P=0.082). The changes differed significantly between the two groups in MAGE [CSII+Lira group = –1.9 (–2.1, –0.3) *vs* CSII group = –0.9 (–1.3, 0.2) mmol/L, P=0.004], and SD [CSII+Lira group = –0.9 (–1.9, –.8) *vs* CSII group = –0.5(–0.8, –0.4) mmol/L, P=0.006] (Supplementary Table S1).

**Figure 4:**
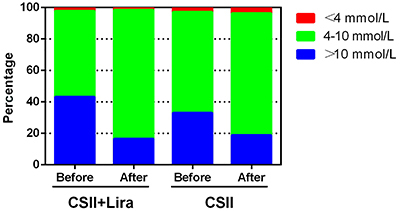
Percentage of time blood glucose was <4.0, 4-10L, and >10 mmol/L at baseline (before) and at the 14-day endpoint (after). CSII: continuous subcutaneous insulin infusion; Lira: liraglutide.

Both interventions significantly decreased the percentage of time spent in the hyperglycemic range (>13.9 mmol/L), but there were no statistically significant differences between the two groups (P=0.458). The percentages of time in the target range (4-10 mmol/L) and >10 mmol/L were increased in both treatment groups, and differences between the groups were statistically significant (P=0.005 and P=0.028, respectively). Ranges of glucose <4.0 mmol/L and hypoglycemia (<3.3 mmol/L) identified by FSL-pro did not differ between regimens (P=0.410 and P=0.274, respectively). Results are summarized in Figure 4 and Supplementary Table S1. There was no hypoglycemia requiring medical assistance in either group throughout the study duration.

### Cardiometabolic risk markers

The serum adiponectin and HO-1 levels were significantly increased from baseline to endpoint in the CSII+Lira group compared to the CSII group [33.54 (3.56, 47.74) *vs* 10.10 (–18.4, 21.97) pg/mL, P=0.013 for adiponectin; 0.46 (–0.02, 1.85) *vs* 0.00 (–0.16, 0.58) ng/mL, P=0.049 for HO-1, respectively]. In the CS1+Lira group, significant decreases from baseline were found for serum leptin [-2.82(3.91) *vs* 1.44 (2.67), P<0.001]. In contrast, no difference was seen for changes in serum IL-6 or 8-iso-PGF2α between these two groups (Table 3).

**Table 3:** Comparison of cardiometabolic risk biomarkers between diabetes mellitus patients under continuous subcutaneous insulin infusion (CSII) and CSII+liraglutide (Lira) treatments.

Variable	CSII+Lira	CSII	P value (between groups)
HO-1 (ng/mL)			
Before	2.0 (1.3, 2.5)	2.3 (2.1, 2.6)	0.188
After	2.9 (1.6, 3.6)	2.5 (2.1, 2.8)	0.237
P value	0.001	0.229	–
Change	0.4 (–0.0, 1.8)	0.0 (–0.2, 0.6)	**0.049**
Leptin (pg/mL)			
Before	27.0 (25.1, 29.9)	26.9 (23.7, 31.2)	0.806
After	23.7 (22.4, 26.9)	28.8 (27.3, 31.4)	**<0.001**
P value	0.000	0.012	–
Change	–2.8 (3.9)	1.4 (2.6)	**<0.001**
Adiponectin (pg/mL)			
Before	134.7 (95.2,156.9)	135.2 (119.7, 157.7)	0.806
After	161.9 (93.3, 195.1)	144.4 (111.5, 154.6)	0.368
P value	0.003	0.516	–
Change	33.5 (3.5, 47.7)	10.1 (–18.4, 22.0)	**0.013**
IL-6 (pg/mL)			
Before	7.6 (1.6)	7.2 (2.8)	0.484
After	6.4 (4.3, 9.8)	7.0 (7.0, 8.5)	0.395
P value	0.155	0.710	-
Change	–1.2 (–2.6, 0.8)	0.0 (–2.1, 0.9)	0.295
8-iso-PGF2a (pg/mL)			
Before	174.7 (171.0, 175.3)	173.5 (171.2, 174.1)	0.051
After	172.4 (169.0, 173.0)	171.8 (165.4, 172.4)	0.093
P value	0.001	0.000	–
Change	–1.9 (3.1)	–3.2 (3.4)	0.137

Data are reported as median (IQR) or mean (SD). Before: before therapy (baseline); After: after 14 days of therapy; HO-1, heme oxygenase-1; IL-6: interleukin-6; 8-iso-PGF2α: 8-iso prostaglandin F2α. Groups were compared by independent *t*-tests or Mann-Whitney U-tests. The paired *t*-tests or Wilcoxon rank sum test were performed to compare the changes before and after intervention in the same group. P values <0.05 are highlighted in bold.

## Discussion

This study demonstrated that in poorly controlled T2DM on a background regimen of metformin and MDI therapy, both CSII and liraglutide therapy showed increased efficacy in glycemic control, and the addition of liraglutide to CSII therapy conferred greater benefits than CSII alone.

The main benefit observed in this study was the reduction of GV. Acute glucose fluctuations are closely related to oxidative stress, inflammation, and endothelial dysfunction, factors traditionally linked to the pathogenesis of vascular damage (19). Previous studies have reported that glycemic fluctuation was associated with both macro- and microvascular events (20). Less frequent monitoring of blood glucose levels using SMBG could not alert a hypoglycemic event and may lead to inaccurate GV assessment, suggesting a lack of or a reduction in glucose fluctuations (21). In contrast, CGM provides a better measurement of the extent of glycemic fluctuation and overall trends by providing assessments at multiple time-points (22). However, these systems are expensive and require constant calibration. Each evaluation was extended only for 3 days, which may be too limited a time interval to fully assess clinical outcomes of interest.

In this study, we used an FGM system to obtain blinded AGP data on glucose over a 14-day period at multiple time points. The newer FGM system, which can comprehensively analyze glucose profiles, offers an alternative glucose monitoring strategy. It has some apparent advantages over other blood glucose monitoring systems. The sensor is small, light, and in most cases stays in place for 14 days. Therefore, when you scan you not only get a glucose reading, but can also see whether your blood glucose levels are starting to go up, down, or are stable. It is more affordable than conventional CGM devices and does not require calibration. The software allows for a consolidated overview of blood glucose levels and quantification of measured glucose exposure, stability, and variability. The reliability of the FGM system has been evaluated in many studies (12). This system is used to explore several aspects of daily glycaemia patterns.

The results of our study showed that both liraglutide and CSII treatment were associated with lower glucose variability than MDI, which agreed with previous studies (13,23). CSII in combination with liraglutide showed a significant improvement in MAGE, CV, and SD during 14 days compared with CSII therapy alone. This might demonstrate that adding liraglutide to CSII therapy could result in greater improvement in GV compared with CSII therapy alone. Prospective studies of GLP-1 combination with insulin have shown either superior or equivalent efficacy in blood glucose control compared with insulin therapy alone (24). We evaluated five blood glucose ranges, which were associated with therapy outcomes relevant for patient management. During a 14-day hospitalization period, the percentages of time when blood glucose was in 4–10, >10, and >13.9 mmol/L ranges were improved in both treatment groups, and the changes in blood glucose ranges in 4–10 and >10 mmol/L were higher in the liraglutide combined therapy than in CSII therapy alone. This result indicated that, compared with CSII alone, CSII in combination with liraglutide treatment enabled a more favorable balance between glucose values in clinically relevant ranges. In this study, the safety of both regimens was further supported by the absence of episodes of hypoglycemia requiring medical assistance.

The combination of GLP-1 and insulin is receiving great attention today as a possible therapeutic choice for the management of T2DM. In a prospective randomized study, exenatide added to metformin and basal insulin therapy significantly reduced weight, GV, and some cardiovascular risk markers while maintaining equivalent HbA1c levels versus a rapid-acting insulin analog regimen (25). Liraglutide, a GLP-1 analog with a 97% homology with endogenous GLP-1, has been demonstrated to improve endothelial function, influence various cellular pathways including anti-inflammatory pathways, and possibly decrease oxidative stress generation. Moreover, it reduced body weight, waist circumference, and blood pressure, which are critical risk factors for cardiovascular disease (26). Specific clinical trials are needed to confirm the usefulness of this therapeutic possibility.

In the present study, we used liraglutide as an add-on therapy to CSII in a 14-day hospitalization period. Most subjects (90%) in the liraglutide plus CSII group lost weight, with a mean weight loss of 2.5 (0.5) kg. Our study supported previous research that showed that liraglutide improved weight reduction (23–26). Furthermore, it has also been shown that even short-term use of liraglutide is associated with weight loss (27). During hospital admission, the subjects received 1400–1800 kcal meals depending on their standard body weight. Therefore, the improvement in body weight may be related to the central and peripheral effects of liraglutide therapy.

Patients with T2DM with high insulin demand have severe insulin resistance and are at high risk of cardiovascular disease. In recent years, the effects of anti-diabetic drugs on cardiometabolic risk factors have gained increasing attention (28). The evidence that a combination of insulin and GLP-1 analog cannot only improve metabolic control but also have a favorable impact on cardiometabolic risk factors and biomarkers of diabetic patients is certainly of great interest. We found that liraglutide increased serum adiponectin levels and reduced serum leptin levels, consistent with previous observations (27,29). Research has shown that GLP-1 could increase adiponectin mRNA content and promote adiponectin secretion via the protein kinase A pathway in 3T3-L1 adipocytes (30). Leptin is the prototypical adipokine, which has been described as a potent modulator of the response to dynamic meal-related signals, which are mediated by GLP-1 receptor. The possibility that GLP-1 might reduce the production of leptin by adipocytes remains to be confirmed, but it is a possible mechanism related to the weight loss of these patients. A randomized, placebo-controlled, crossover study suggested that a short course of liraglutide therapy did not change body weight significantly, but there was a significant decrease in the percentage change of leptin while on liraglutide therapy compared with the placebo (27). However, short-term effects in response to early liraglutide therapy include an increase in gastric inhibitory peptides (GIPs), which may promote the anorexigenic actions of liraglutide, whereas compensatory decreases of leptin may counteract the effects of liraglutide (27). The corresponding changes of adiponectin and leptin levels could be coupled with a decrease in insulin resistance. HO-1 is a microsomal enzyme that can suppress oxidative stress. Some studies have shown that the anti-oxidant properties of liraglutide may be related to the restoration of HO-1 (31). Oeseburg H. et al (32) revealed that GLP-1 can induce the expression of oxidative defense genes HO-1 and NQO1 in endothelial cells. Our study showed that liraglutide increased the serum concentrations of HO-1, which might indicate an increase in anti-oxidant capacity. Previous literature has demonstrated that GLP-1 could enhance NRF2, consequently increasing HO-1 expression against diabetic-mediated oxidation and inflammation (33,34). Because adiponectin, leptin, and HO-1, as well as weight, are all associated with cardiometabolic risks, these findings suggest that treatment with liraglutide might lead to medical benefits.

Serum levels of IL-6 and 8-iso-PGF2a were used to measure the status of inflammatory and oxidative stress in the body in the current study. Some studies have suggested that the combination of insulin and GLP-1 is more effective than insulin alone in improving inflammation and oxidative stress in T2DM. The levels of 8-iso-PGF2α in the two groups were significantly lower after treatment, suggesting that control of blood glucose and reduction of blood glucose fluctuations can reduce the level of oxidative stress in diabetic patients. However, there was no significant difference in the IL-6 levels and the changes in 8-iso-PGF2α levels between the two groups, which may be related to the shorter application time of liraglutide. It is also suggested that the application of liraglutide in the short term has no obvious anti-inflammatory effect.

Our study evaluated the use of the FSL-pro for a 14-day period in a clinical environment. Information provided during a single wearing period is considered by most clinicians to be of therapeutic assistance to most patients, and AGP analysis provides a simple means to access and interpret the data captured by the system. Strengths of our study included a direct comparison of two treatment regimens, attainment of a high rate of adherence to the protocol, direct demonstration of a reduction in GV using a FGM system, and confirmation of the safety and efficacy of the newer method of glycemic monitoring and treatment.

However, the present study has several limitations. First, it is a single-center study with a relatively small sample size, which may limit the statistical power when analyzing some clinical parameters. In addition, the study period was only 14 days, which may not have been sufficient to assess the additional benefits associated with GV. We need larger prospective studies with longer duration on the combination of CSII and liraglutide and potentially a multicenter research study to further validate our findings.

In conclusion, the addition of liraglutide to CSII therapy was superior to CSII alone in improving GV, weight, and some cardiometabolic biomarkers in patients with T2DM. The FGM system is a novel and useful method for glucose monitoring. The safety and benefits of this therapy are still subject to large-scale, longer-duration prospective studies.

## Supplementary Material

Click here to view [pdf].
